# Interpretable Multihorizon Glucose Forecasting for Assessing Nutritional Information Impact in Type 1 Diabetes Management: Model Development and Validation Study

**DOI:** 10.2196/105049

**Published:** 2026-09-21

**Authors:** Carlos Gallardo-García, M Elena Hernando, David Subías, Mercedes Rigla, Gema García-Sáez

**Affiliations:** 1 Bioengineering and Telemedicine Group, Centro de Tecnología Biomédica, ETSI de Telecomunicación, Universidad Politécnica de Madrid, Avda Complutense 40, Madrid, 28040, Spain, 34 910672474; 2 Instituto de Investigación Hospital 12 de Octubre (imas12), Research Institute Hospital 12 de Octubre, Madrid, Madrid, Spain; 3 Biomedical Research Networking Center in Bioengineering, Biomaterials and Nanomedicine, Madrid, Spain; 4 Endocrinology and Nutrition Department, Parc Tauli Sabadell University Hospital, Institute of Research and Innovation Parc Tauli, Sabadell, Catalonia, Spain; 5 Universitat Autònoma de Barcelona, Barcelona, Catalonia, Spain

**Keywords:** temporal fusion transformer, attention model, explainable artificial intelligence, type 1 diabetes, nutritional information, clinical decision-support, glucose forecasting, deep learning, multihorizon prediction

## Abstract

**Background:**

Type 1 diabetes is characterized by absolute insulin deficiency, requiring exogenous insulin therapy to maintain blood glucose levels within safe ranges. Postprandial glucose control remains particularly challenging, and current meal-related strategies are mainly based on carbohydrate intake. However, other macronutrients, such as fats and proteins, may also influence the magnitude and timing of the glycemic response and are not usually incorporated into glucose forecasting models.

**Objective:**

This study aims to develop and evaluate multihorizon blood glucose forecasting models to assess the impact of incorporating detailed nutritional information, with particular emphasis on the postprandial period and to analyze how the contribution of different nutrients varies across prediction horizons.

**Methods:**

We trained temporal fusion transformer (TFT) models on continuous glucose monitoring, insulin, and meal data with different combinations of nutritional variables from 351 adults with type 1 diabetes (15,601 meals) to predict glucose values up to 4 hours ahead. Carbohydrates were used as the baseline nutritional input, and additional configurations included fats, proteins, sugars, and complex carbohydrates. Model performance was evaluated globally and in predictions initiated at meal intake. Prediction horizons were grouped into early and late intervals, corresponding to 0-2 hours and 2-4 hours, respectively. The interpretability mechanisms of the TFT and integrated gradients method were used to analyze the relative contribution of nutritional variables.

**Results:**

Models incorporating additional nutritional information generally outperformed the carbohydrate-only baseline. In the global analysis, the combination of carbohydrates, fats, and proteins achieved the best performance in the early prediction interval, reducing mean root mean squared error (RMSE) and mean absolute error (MAE) from 30.48 mg/dL and 21.02 mg/dL to 29.23 mg/dL and 20.15 mg/dL. At late intervals, distinguishing between complex carbohydrates and sugars, together with fats and proteins, provided the best performance, reducing mean RMSE and MAE from 45.34 mg/dL and 34.73 mg/dL to 43.24 mg/dL and 32.65 mg/dL. A similar temporal dependency pattern was observed in the postprandial evaluation and was partially supported by the interpretability analysis.

**Conclusions:**

These findings suggest that incorporating a more comprehensive representation of meal composition may modestly improve postprandial glucose forecasting. Horizon-specific attribution patterns indicated that the models used nutritional inputs differently across the forecast horizon; however, these findings should not be interpreted as causal or physiological effects of individual nutrients. Further external and prospective validation is required before the potential clinical utility of these models can be assessed.

## Introduction

Type 1 diabetes mellitus (T1D) is a chronic disease characterized by an absolute insulin deficiency, generally resulting from the autoimmune destruction of pancreatic β cells. Consequently, patients require exogenous insulin therapy to maintain glucose levels within safe ranges and reduce the risk of acute and chronic complications [1]. However, glycemic control in T1D remains challenging due to high intra- and interindividual variability, as well as the simultaneous influence of multiple physiological and behavioral factors, including insulin administration, physical activity, stress, sleep, and food intake [2].

Postprandial glycemic response represents one of the main challenges in the daily management of T1D. Traditionally, meal planning and prandial bolus calculation have been mainly based on the amount of carbohydrate intake. Nevertheless, growing evidence suggests that other nutritional components also modulate glucose dynamics [3-5]. In particular, proteins may contribute to delayed glucose elevations through gluconeogenesis, whereas fats may slow gastric emptying, delay the glycemic peak, and induce transient insulin resistance [3]. Likewise, factors such as fiber, total energy load, or the overall meal composition may alter the magnitude and duration of the postprandial response [6,7]. Therefore, a more comprehensive nutritional characterization could provide relevant information to improve glucose prediction, especially over extended time horizons.

In recent years, artificial intelligence, and particularly deep learning techniques, have gained an increasingly prominent role in glucose prediction for patients with T1D [8]. These models enable the capture of nonlinear relationships and complex temporal dependencies from continuous glucose monitoring signals, insulin records, physical activity, and other contextual data. However, despite the advances achieved, accurate medium- and long-term glucose prediction remains an open problem, particularly during the postprandial period, where the interaction between insulin, meal composition, and previous glycemic state may lead to highly heterogeneous responses [4].

Recently, architectures based on attention mechanisms have become increasingly important for time-series modeling. Since their introduction in machine translation tasks [9] and their consolidation with transformers [10], attention mechanisms have demonstrated a strong ability to model long-range dependencies in time series. In the field of T1D, they have been applied to problems such as nocturnal hypoglycemia prediction [11] or prandial bolus optimization [12]. Zhu et al [13] introduced an edge-based transformer architecture for multihorizon glucose prediction and subsequently extended transformer-based prediction to different populations [14]. Glucose Prediction Transformer (GPFormer; Zhu et al [15]) further addressed multihorizon glucose forecasting and cross-population generalization. Multihead attention has also been incorporated into neural basis expansion architectures, such as Neural-Basis Expansion Analysis Time Series Forecasting (N-BEATS) [16]. Moreover, foundational models, such as TimeGPT (Nixtla), have been evaluated in patients with T1D [17]. Among these architectures, the temporal fusion transformer (TFT) is particularly attractive, as it combines high predictive performance with internal interpretability mechanisms that allow the relative importance of static and dynamic variables in the prediction process to be estimated [18]. In glucose forecasting, Karagoz et al [19] conducted a comparative evaluation of several transformer-based multihorizon architectures, like Patch Time Series Transformer (PatchTST) or Crossformer, and reported competitive performance across different horizons. Although TFT was not included in that comparative study, Zhu et al [13] separately reported that TFT achieved lower RMSE values than other recent deep learning approaches, such as Neural Hierarchical Interpolation for Time Series Forecasting (N-HiTS) or N-BEATS, in multihorizon blood glucose prediction.

Nevertheless, the literature devoted to the explainability of glucose prediction models remains limited, especially when analyzing the model’s dependence on nutritional components. Most studies have focused on variables such as previous glucose, insulin, and total carbohydrate intake [20], whereas the impact of a broader nutritional characterization has been less extensively explored. In this regard, Annuzzi et al [5] initially evaluated the effect of incorporating carbohydrates, proteins, fats, and fiber into postprandial prediction models at horizons ranging from 15 to 60 minutes. Subsequently, they extended this analysis [21] by using Shapley additive explanations (SHAP) [22,23] to study the individual contribution of different nutritional factors in neural models with prediction horizons (PH) of 15, 60, and 120 minutes.

Other recent studies have also incorporated explainability techniques into the analysis of postprandial glycemic responses. Xiong et al [24] used SHAP to interpret personalized models based on continuous glucose monitoring (CGM) data, insulin, clinical characteristics, and meal macronutrient composition, with the aim of estimating the overall postprandial glycemic response. Rehman et al [25], in turn, used SHAP and local interpretable model-agnostic explanations to explain classification models for hypoglycemia and hyperglycemia events within a postprandial window of up to 4 hours.

However, to our knowledge, some relevant aspects are not jointly addressed in the existing literature, including continuous multihorizon prediction with detailed nutritional information, and the interpretable analysis of its influence over extended PHs. In this work, we use a TFT model as a multihorizon framework for glucose prediction in adults with T1D, explicitly designed to evaluate how different macronutrient representations influence forecasts across 8 PHs up to 4 hours. The model is evaluated both globally and specifically during the postprandial period to analyze how detailed meal composition (carbohydrates, fats, proteins, sugars, and complex carbohydrates; CC) contributes to short- and long-term glucose evolution. In addition, we combine aggregated variable selection network (VSN) weights from TFT with horizon-specific integrated gradients to obtain complementary, horizon-dependent estimates of the relative contribution of nutritional variables and their interaction with other relevant signals. Thus, the proposed framework enables a controlled evaluation of whether detailed meal composition provides additional predictive information beyond carbohydrates and offers complementary insight into how the trained models use nutritional inputs across forecast horizons. These interpretability results are intended to characterize model behavior rather than establish causal or physiological effects. The findings may inform future research on nutrition-aware glucose forecasting and the development of interpretable prediction models.

## Methods

### Dataset

In this study, we used the type 1 diabetes and exercise initiative (T1-DEXI) dataset, which comprises 497 adults with T1D who participated in structured home-based aerobic, interval, or resistance exercise programs over four-week periods [26]. The mean age of the participants was 37 (SD 14) years, with 162 subjects assigned to the aerobic exercise group, 165 to the interval exercise group, and 170 to the resistance exercise group.

T1-DEXI is one of the largest and most comprehensive real-world cohorts available in this field. It integrates information from multiple sources, including CGM, insulin administration, dietary intake, physical activity, sleep quality, and demographic information. This multimodal richness enables the study of glucose dynamics under real-life conditions and allows the joint influence of different physiological and behavioral factors to be analyzed.

A particularly relevant aspect of the T1-DEXI study is the detailed recording of dietary intake. Dietary energy and nutrient intake were measured using the remote food photography method implemented in a dedicated mobile application. Participants used the application to photograph all meals, record estimated carbohydrate content, identify the meal type, and optionally add a free-text description. The application transmitted food images to a custom software system, the food photography application, where they were stored for subsequent analysis. Based on previously described and validated methods [27,28], energy and nutrient intake were estimated by comparing participants’ food images with images of foods of known portion size contained in a searchable archive. This archive provides standardized portion images for portion-size estimation and links each item to the Food and Nutrient Database for Dietary Studies, the producer’s nutrient information, or a custom recipe for energy and nutrient calculation. Image analysis is conducted through a semiautomated process that combines human ratings with computer-assisted methods. A reference card is included in each image to standardize color and perspective, facilitating analysis by both human raters and automated image-processing algorithms. After the images are received by the server, a trained human rater uses a computer-assisted interface to identify the foods depicted and map them to the appropriate nutrient entries in the database

Unlike traditional manual records, this approach provides more complete and structured dietary information beyond total carbohydrate intake, including macronutrient composition such as fats, proteins, and sugars. Therefore, these data allow the model to incorporate richer meal-related variables and to analyze their contribution to future glucose prediction, especially during the postprandial period.

### Preprocessing

CGM values were constrained to the 40‐400 mg/dL interval, with out-of-range values clipped to the nearest boundary. For input variables, short gaps in CGM (less than 30 minutes) were handled differently in the training and evaluation sets: in the training set, missing input CGM values were imputed using linear interpolation, whereas in the validation and test sets they were imputed using a last-observation-carried-forward (LOCF) approach to avoid introducing information from future glucose measurements. Gaps longer than 30 minutes were not imputed. Instead, each participant’s time series was divided into contiguous segments at these gaps, and sliding windows were generated separately within each segment, preventing any window from spanning a long gap. The total amount of imputed CGM data accounted for 2.96%, 3.96%, and 4.38% of the remaining data after long gaps were removed in the training, validation, and test partitions, respectively. For the prediction targets, missing CGM values were not imputed. During testing, any window in which one or more target CGM values were missing was excluded from the evaluation, ensuring that forecast performance was assessed only against observed glucose measurements.

In this study, we distinguished between scheduled basal insulin (the basal rate profile prescribed by the endocrinologist and programmed in the pump) and actual delivered basal insulin (including temporary basal adjustments and suspensions), and both were used as predictive variables. Basal insulin deliveries from insulin pumps were resampled at 5-minute intervals based on pump-reported basal rates. Insulin boluses and meal intakes were encoded as time-stamped events on the 5-minute sampling grid. For each meal, the estimated macronutrient amounts were assigned to the time step corresponding to the meal timestamp and were included as event-based covariates in the input sequence. Thus, carbohydrates and the additional nutrient variables were modeled as instantaneous exogenous inputs rather than as continuously updated signals. When multiple boluses or meal samples occurred within the same interval, their total amount was summed. For square-wave boluses, the total insulin dose was uniformly redistributed into 5-minute intervals over the programmed bolus duration.

### Dataset Construction and Partitioning

**Figure 1.**
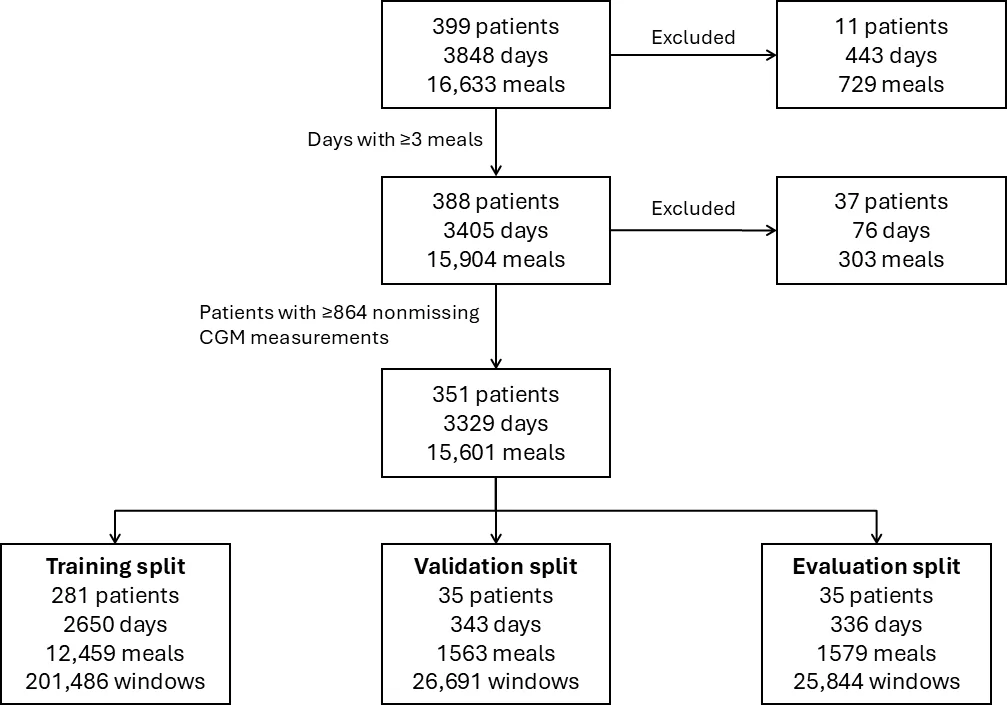
Schematic description of the cohort selection, data filtering, and dataset partitioning for the type 1 diabetes and exercise initiative dataset. Type 1 diabetes and exercise initiative is a real-world, home-based exercise study for adult US residents with type 1 diabetes. The flow diagram summarizes the application of inclusion and exclusion criteria to continuous glucose monitoring and dietary records. Days with fewer than three meals were removed; only individuals with 864 or more nonmissing CGM measurements were included. The remaining cohort consisted of 351 participants, 3329 days, and 15,601 meals. This cohort was split into training, validation, and test sets used to develop and evaluate the glucose forecasting models.

The data filtering and cohort selection process is summarized in Figure 1. Days with fewer than 3 recognition-based meal events were excluded to ensure sufficient dietary information density and reduce bias from incomplete records. A meal event was operationally defined as a dietary record containing a value greater than zero for at least one of the following variables: carbohydrates, fats, proteins, or sugars. At the patient level, only individuals with at least 864 nonmissing CGM measurements were retained, equivalent in number to 3 complete days of valid data (and ≥3 meals/day). This threshold was applied to ensure adequate longitudinal representation. After applying these criteria, the final dataset comprised 351 patients, 3329 days, and 15,601 meals (mean; SD number of days per patient: 9.48; 2.4).

The dataset was split into training, validation, and test sets using an 80/10/10 patient-wise partition, ensuring that each individual appeared in only one subset to prevent data leakage and to assess inter-patient generalization. The resulting splits are detailed in Figure 1.

Multimedia Appendix 1 summarizes the baseline demographic and treatment characteristics of the final study cohort for the included subsets (training, validation, and test), as well as for participants whose data were excluded from the original dataset. Although some variation in percentage distributions was observed, particularly in the smaller validation and test subsets, the characteristics were broadly comparable across all partitions. No statistically significant differences were observed between the included and excluded participants with respect to sex (*P*=.22), insulin delivery modality (*P*=.16), age group (*P*=.10), racial or ethnicity (*P*=.49), or T1D duration (*P*=.54). However, a statistically significant difference was observed in the distribution of glycated hemoglobin between the included and excluded participants (*P*=.004).

### Architecture and Experiments

Training samples were generated using a sliding-window approach with a 6-hour historical window, a 15-minute stride, and a 4-hour PH. This setup is based on previous works [29], and enables the model to capture both short-term glucose dynamics and the delayed effects of meals and insulin on glycemia [30], while maintaining a balance between temporal resolution and computational cost.

To assess the impact of additional nutritional information, multiple input-variable configurations were evaluated, incorporating different combinations of macronutrients. These included the following configurations: (1) carbohydrates; (2) carbohydrates with fats; (3) carbohydrates with proteins; (4) carbohydrates with fats and proteins; (5) carbohydrates with sugars; (6) carbohydrates with fats, proteins, and sugars; (7) carbohydrates with fats, proteins, and explicit representation of carbohydrate subtypes by distinguishing between simple sugars and CC. CC were not directly provided as a separate variable but were derived for each recorded meal as the difference between total carbohydrates and sugars:


(1) CC (g) = total carbohydrates (g) − sugars (g)

The carbohydrates-only configuration was established as the baseline model, reflecting the predominant approach in both the literature and current clinical systems. This allowed a controlled assessment of the added value of incorporating more detailed nutritional information.

All configurations included CGM data, nutritional variables, actual delivered basal insulin, and prandial boluses as observed input variables (available to the model only within the 6-hour historical encoder period). Scheduled basal insulin was included as a known input variable because it corresponded to the basal profile programmed in advance and was therefore provided across both the encoder and decoder periods. In addition, 2 temporal indices were used. “Time ID” encoded the time of day using 288 five-minute intervals, whereas “Relative Time ID” encoded the sequential position of each observation within each continuous data segment and was reset at the beginning of each segment.

In addition, a conventional long short-term memory (LSTM) model was trained to assess whether the TFT architecture provided additional predictive value over a specialized recurrent neural network designed to process sequential data. The LSTM used the same 6-hour historical window, input variables, data partitions, preprocessing, and 4-hour output horizon as the carbohydrates model configuration. Its architecture comprised one LSTM layer with 128 hidden units, followed by a dropout layer with a rate of 0.2 and a fully connected layer producing 48 future glucose values, corresponding to the 4-hour PH at 5-minute intervals. The model was trained using the mean squared error loss function and the Adam optimizer with a learning rate of 1×10^-3^, a batch size of 128, and a maximum of 100 epochs. Early stopping monitored mean squared error metric with a patience of 10 epochs.

A correlation analysis of nutritional variables was conducted to identify potential redundancies and their impact on model learning.

Hyperparameters and architecture were selected through grid search and kept fixed across all configurations to ensure comparability. The complete hyperparameter space explored is shown in Multimedia Appendix 2. The final TFT architecture comprised 4 attention heads, 64 hidden units, one LSTM layer, and a dropout rate of 0.2. The model was trained using the Adam optimizer with a learning rate of 1×10^–4^. Quantile loss with quantiles (0.25, 0.5, 0.75) was used as the training objective. Training was performed with a batch size of 64 for a maximum of 100 epochs. Early stopping monitored the validation quantile loss with a patience of 10 epochs, and the model parameters corresponding to the lowest validation loss were retained for evaluation. Target glucose was normalized using the EncoderNormalizer (PyTorch Forecasting), based on the historical encoder values available within each prediction window. Each model configuration was trained once using the same fixed random seed.

To address the imbalance between windows containing meal intake and non–postprandial windows, we applied an oversampling strategy exclusively to the training set. A window was classified as postprandial if its prediction origin coincided with a recorded meal or occurred within the subsequent 15 minutes. This interval was selected to accommodate the 15-minute stride used to generate prediction windows and to include the first available forecast origin following each recorded meal. Postprandial windows were assigned a sampling weight of 2, whereas all remaining windows were assigned a weight of 1. The validation and test partitions were not oversampled and retained their original distributions. This strategy was intended to increase the model’s reliance on nutritional inputs while reducing its relative emphasis on non–postprandial glucose dynamics.

Finally, we conducted an additional experiment to assess whether physical activity influenced the relative performance of the nutritional model configurations. In this secondary analysis, we excluded windows in the test set that contained any physical activity events and evaluated the models on the remaining windows only. This experiment was used to compare the best-performing models with the baseline model at each PH.

### Model Evaluation and Interpretability

Model performance was evaluated using the root mean squared error (RMSE), as a standard regression metric, and mean absolute error (MAE) as a complementary metric. Both metrics are defined in equations (2) and (3), respectively.


(2) $$RMSE=\sqrt{\frac{1}{N}\sum_{i=1}^{N}{\left(x_{i}-{\overset{ˆ}{x}}_{i}\right)}^{2}}$$

(3) $$\begin{array}{c} MAE=\frac{1}{N}\sum_{i=1}^{N}|x_{i}-{\overset{ˆ}{x}}_{i}| \end{array}$$

**Figure 2.**
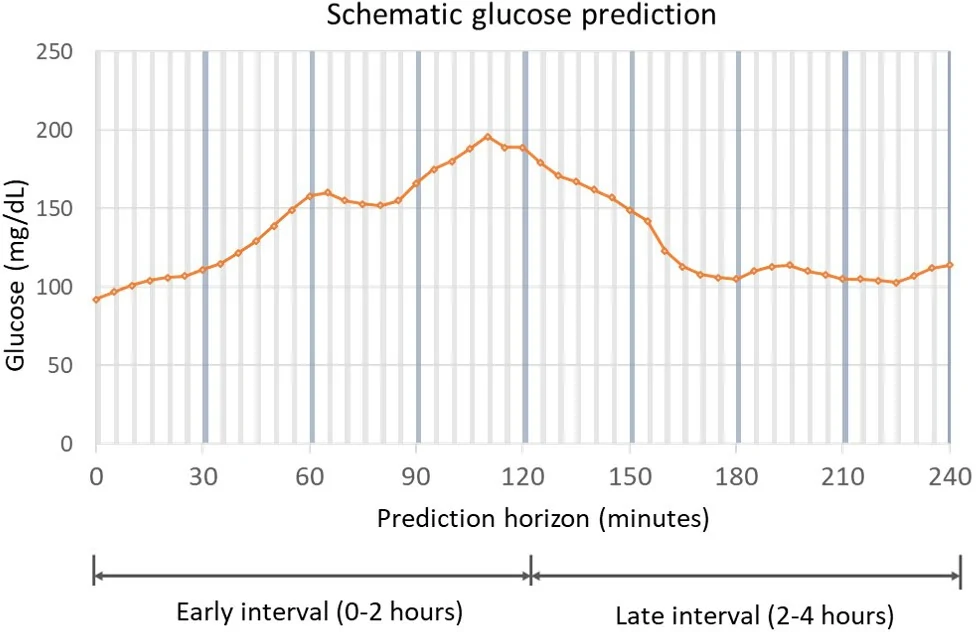
Schematic illustration of the multi-horizon evaluation procedure used for the temporal fusion transformer glucose forecasting models. The illustrative trajectory and prediction interval are not derived from an actual model prediction. The plot shows prediction horizons utilized to assess predictions over a 4-hour prediction window, emphasizing the early (0‐2 hours) and late (2‐4 hours) intervals. These intervals are used to analyze the impact of nutritional variables on postprandial glycemic dynamics.

RMSE and MAE were computed across all PHs (30, 60, 90, 120, 150, 180, 210, and 240) and further averaged over 2 PH intervals: early (0‐2 hours) and late (2‐4 hours). Figure 2 illustrates the definition of multihorizon blood glucose forecasting generated by the TFT model at different PH and for the 2 intervals. Grouping into 2 wider intervals allows a clearer analysis of the effect of nutritional variables on early and late postprandial glucose prediction.

Moreover, this analysis was performed both for the overall dataset and for the subset of predictions initiated around meal intake (postprandial windows). Postprandial windows were not excluded when an additional meal, correction bolus, or physical activity event occurred during the subsequent 4-hour PH. Therefore, this analysis represents postprandial forecasting under free-living conditions rather than glucose responses to isolated meals. Differences between model configurations were evaluated in each analyzed PH, using paired Student *t*-tests. Their uncertainty was expressed using 95% *t*-based CIs for the paired mean differences. *P* values were adjusted for multiple comparisons using the Holm method.

To assess clinical impact, we applied Clarke error grid analysis (EGA) [31], which assigns each reference–prediction pair to zones A-E based on the potential consequences of glucose estimation errors. Zone A and B represent clinically accurate or acceptable predictions, whereas Zones C, D, and E represent errors that could lead to inappropriate clinical decisions. In particular, Zone D includes failures to detect hypoglycemia or hyperglycemia, whereas Zone E includes errors that could lead to treatment in the wrong direction. We compared the model using carbohydrates only and the best-performing model for each PH.

Model interpretability was assessed using the VSN of the TFT model. These layers assign normalized selection weights reflecting the relative relevance of each input variable in the prediction. After model training, the encoder variable weights were extracted and aggregated across samples and encoder time steps for the representative best-performing nutritional configurations. This allowed us to identify the relative contribution of each predictive variable in the selected models.

Additionally, integrated gradient analysis was performed on the best-performing models to assess interpretability at the horizon-specific level. Twenty postprandial test windows were randomly selected per participant for the analysis to ensure balanced participant representation. The baseline was constructed by setting the nutritional inputs at 0g and keeping the rest of the variables unchanged. Integrated gradients were approximated using 32 Gauss-Legendre integration steps. Absolute attributions were aggregated across the encoder time steps and averaged across the selected windows. At each PH, they were subsequently normalized across the nutritional variables to obtain relative attribution percentages summing to 100%. These attributions describe the dependence of model predictions on the nutritional inputs and should not be interpreted as causal, independent, or physiological effects, particularly given the correlations among nutrients.

### Hardware and Efficiency

All experiments were conducted using Nvidia GeForce RTX 3090 GPU with 24 GB of VRAM, an AMD Ryzen 9 5950X CPU, and 125 GB of RAM. The models were implemented using Python 3.13.5 (Python Software Foundation), PyTorch 2.7.1 (PyTorch Foundation), PyTorch Forecasting 1.4.0 (Jan Beitner and contributors), and Lightning 2.5.2 (Lightning AI). Inference time corresponds to a range of 2.4‐2.6 minutes per prediction window.

### Ethical Considerations

This work is a secondary analysis of deidentified data from the T1-DEXI study. The T1-DEXI study protocol and all study-related materials were reviewed and approved by the Jaeb Center for Health Research Institutional Review Board (IRB), and all participants provided electronic informed consent prior to participation. The corresponding IRB approval number was not reported in the study documentation available to the authors and is not available for inclusion in this manuscript. The original consent included permission for future secondary research using deidentified data without requiring additional consent. The present study involved no new recruitment, participant contact, intervention, or additional data collection. No compensation was provided for this secondary analysis. Information about any compensation provided in the primary T1-DEXI study was not available to the authors.

Upon study completion, deidentified study data were transferred to and stored on the Vivli data-sharing platform (ID: T1-DEXI; [32]) for access by qualified researchers.

## Results

**Figure 3.**
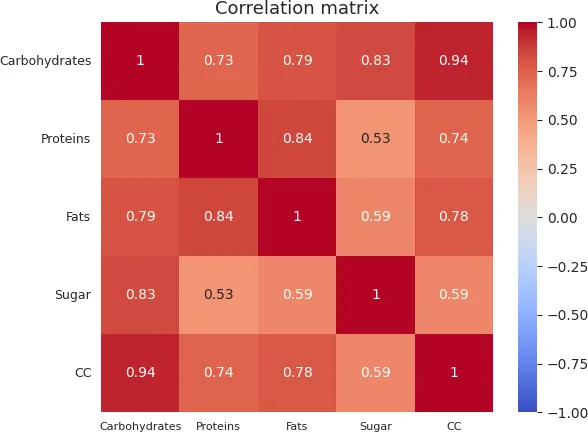
Correlation matrix of nutritional variables used in the temporal fusion transformer models trained on continuous glucose monitoring, insulin, and meal data from adults in the type 1 diabetes and exercise initiative study. The heatmap presents Pearson correlations among nutritional inputs (total carbohydrates, fats, proteins, sugars, and complex carbohydrates), illustrating the strong associations between macronutrients that may influence model learning and the interpretation of variable-importance estimates across the 0‐4-hour postprandial prediction horizons.

The correlation analysis among the predictor variables (see Figure 3) revealed a strong association between carbohydrates, fats, and proteins, as well as between carbohydrates and sugars. This pattern likely reflects the fact that larger meals tend to contain higher amounts of multiple macronutrients. The high correlation between carbohydrates and sugars is expected, given that sugars constitute a fraction of the total carbohydrate content of the meal.

Table 1 summarizes model performance of the conventional LSTM model and TFT configurations with different nutritional variables across PH. The manually configured LSTM model produced higher prediction errors than the carbohydrates model configuration despite using the same input variables. However, because the LSTM was not hyperparameter-optimized using the same procedure as the TFT, this finding should be interpreted as a descriptive comparison with a recurrent baseline. Overall, incorporating additional nutritional variables reduced the RMSE compared with the carbohydrates-only baseline in most scenarios. The baseline model showed higher errors across nearly all PHs, with minor exception at the shortest horizon (30 minutes), where three extended configurations slightly underperformed. From 60 minutes onward, all models incorporating additional nutritional information consistently outperformed the baseline.

**Table 1.** Global participant-level root mean squared error (RMSE) obtained for each combination of nutritional variables across the different prediction horizons, reported as mean (SD) mg/dL.

Nutrients	PH^a^ (minutes)							
	30	60	90	120	150	180	210	240
LSTM^b^	20.11 (4.5)	32.25 (7.4)	39.23 (9.1)	43.07 (10.0)	45.09 (10.3)	46.28 (10.4)	46.94 (10.7)	47.32 (10.8)
Carbohydrates (mg/mL), mean (SD)	18.99 (4.7)	30.40 (8.0)	37.57 (10.2)	41.94 (11.8)	44.47 (12.7)	45.70 (13.3)	46.22 (13.6)	46.56 (13.6)
Carbohydrates+fats (mg/mL), mean (SD)	18.87 (4.5)	29.68 (7.6)	36.32 (9.7)	40.24 (10.9)	42.55 (11.6)	43.94 (12.2)	44.77 (12.6)	45.29 (12.8)
Carbohydrates+proteins (mg/mL), mean (SD)	19.02 (4.5)	29.93 (7.6)	36.51 (9.5)	40.31 (10.5)	42.56 (11.1)	43.93 (11.6)	44.80 (11.9)	45.42 (12.1)
Carbohydrates+sugars (mg/mL), mean (SD)	18.91 (4.5)	29.56 (6.6)	36.17 (9.5)	40.08 (10.6)	42.32 (11.4)	43.69 (11.9)	44.56 (12.2)	45.21 (12.5)
Carbohydrates+fats+proteins (mg/mL), mean (SD)	18.53 (4.4)	29.22 (7.5)	35.95 (9.6)	40.05 (10.8)	42.47 (11.4)	43.95 (11.9)	44.83 (12.1)	45.45 (12.3)
Carbohydrates+fats+proteins+sugars (mg/mL), mean (SD)	19.05 (4.6)	29.88 (8.0)	36.50 (10.2)	40.37 (11.6)	42.58 (12.4)	43.87 (12.8)	44.52 (13.0)	44.86 (13.3)
^c^CC^c^+fats+proteins+sugars (mg/mL), mean (SD)	19.00 (4.6)	29.77 (7.9)	36.35 (10.1)	40.10 (11.4)	42.30 (12.0)	43.51 (12.4)	44.14 (12.6)	44.55 (12.9)

a PH: prediction horizon.

b LSTM: long short-term memory.

c CC: complex carbohydrates.

Model performance varied according to PH. In early horizons, between 30 and 120 minutes, the model trained with carbohydrates, fats, and proteins (carbohydrates+fats+proteins) consistently achieved the lowest errors. At the intermediate 120-minute horizon, performance differences between configurations became less pronounced. At longer horizons (≥150 minutes), the best performance was achieved by models incorporating carbohydrate subtypes, specifically the combination of CC, fats, proteins, and sugars.

Table 2 summarizes model performance by grouping the PHs into early (first two hours of the PH) and late (between 2 and 4 hours) intervals.

**Table 2.** Global patient-level RMSE^a^ and MAE^b^ obtained after grouping the PHs^c^ into the 0-2 and 2- to 4-hour intervals, reported as mean (SD) mg/dL.

Nutrients	PH=0-2 hours		PH=2-4 hours	
	RMSE (mg/dL), mean (SD)	MAE (mg/dL), mean (SD)	RMSE (mg/dL), mean (SD)	MAE (mg/dL), mean (SD)
Carbohydrates	30.48 (8.0)	21.02 (5.5)	45.34 (13.1)	34.73 (10.3)
Carbohydrates+fats	29.56 (7.6)	20.45 (5.2)	43.69 (12.1)	33.40 (9.5)
Carbohydrates+proteins	29.72 (7.5)	20.56 (5.1)	43.72 (11.5)	33.42 (9.2)
Carbohydrates+sugars	29.46 (7.5)	20.41 (5.1)	43.49 (11.8)	33.27 (9.1)
Carbohydrates+fats+proteins	29.23 (7.5)	20.15 (5.1)	43.69 (11.7)	33.42 (9.0)
Carbohydrates+fats+proteins+sugars	29.74 (8.0)	20.41 (5.5)	43.57 (12.7)	32.95 (10.1)
CC^d^+fats+proteins+sugars	29.61 (8.0)	20.30 (5.4)	43.24 (12.3)	32.65 (9.8)

a RMSE: root mean squared error.

b MAE: mean absolute error.

c PH: prediction horizon.

d CC: complex carbohydrates.

The aggregated results confirm the trend observed in the individual horizon analysis. In the early interval, the carbohydrates+fats+proteins model achieved the best performance, with an RMSE of 29.23 mg/dL and an MAE of 20.15 mg/dL, corresponding to an improvement over baseline of approximately 4% in both metrics.

In the late interval, the best performance was achieved by the CC+fats+proteins+sugars model (RMSE 43.24 mg/dL; MAE 32.65 mg/dL), representing relative improvements of 4.6% and 6.0% in RMSE and MAE, respectively, compared with the carbohydrate-only model. Notably, error reductions were more pronounced in the late interval.

Results obtained from predictions initiated at mealtime or up to 15 minutes afterward (Table 3) showed a similar pattern. LSTM again produced higher errors than the C model. At the 30-minute PH, some configurations slightly underperformed the baseline; however, from 90 minutes onward, all models incorporating additional nutritional information reduced prediction error.

**Table 3.** Postprandial patient-level root mean squared error obtained for each combination of nutritional variables in predictions initiated at the time of meal intake or up to 15 minutes afterward.

Models	PH^a^							
	30	60	90	120	150	180	210	240
LSTM^b^	25.46 (9.8)	41.98 (13.5)	48.95 (13.8)	50.47 (13.2)	50.64 (14.2)	50.36 (14.8)	49.54 (14.6)	49.11 (13.2)
Carbohydrates (mg/dL), mean (SD)	24.03 (9.3)	38.84 (11.1)	45.58 (12.8)	47.94 (13.7)	49.22 (15.4)	49.20 (16.9)	49.40 (16.2)	49.02 (15.3)
Carbohydrates+fats (mg/dL), mean (SD)	24.05 (8.7)	38.19 (10.3)	44.40 (12.9)	46.33 (13.3)	47.18 (14.3)	47.33 (15.2)	48.19 (15.0)	47.91 (14.3)
Carbohydrates+proteins (mg/dL), mean (SD)	24.45 (9.0)	38.74 (10.8)	44.65 (12.8)	46.75 (13.3)	47.66 (14.5)	48.07 (15.3)	48.51 (14.9)	48.15 (14.0)
Carbohydrates+sugars (mg/dL), mean (SD)	23.78 (8.4)	37.86 (10.7)	43.91 (12.5)	45.93 (13.2)	46.79 (13.9)	47.44 (14.7)	48.33 (14.5)	47.93 (13.9)
Carbohydrates+fats+proteins (mg/dL), mean (SD)	23.29 (8.6)	37.40 (10.2)	43.87 (11.9)	46.01 (12.6)	47.03 (13.6)	47.56 (14.8)	48.11 (14.1)	48.17 (13.8)
Carbohydrates+fats+proteins+sugars	24.47 (7.2)	39.25 (11.4)	44.52 (13.9)	46.00 (14.2)	46.93 (15.8)	47.46 (16.8)	48.15 (16.6)	47.14 (15.9)
CC^c^+fats+proteins+sugars	24.47 (8.8)	38.85 (10.7)	44.20 (13.5)	45.64 (13.7)	46.36 (15.5)	47.13 (16.1)	47.40 (15.6)	46.12 (14.9)

a PH: prediction horizon.

b LSTM: long short-term memory..

c CC: complex carbohydrates.

In this postprandial scenario, the carbohydrates+fats+proteins configuration achieved the lowest RMSE in early PHs (30‐90 min), whereas the CC+fats+proteins+sugars model performed best at longer horizons (120‐240 min).

When aggregating horizons into early and late intervals (Table 4), the same trend was observed: carbohydrates+fats+proteins yielded the lowest errors in the 0‐2 hour interval, while CC+fats+proteins+sugars achieved the best performance in the 2‐4 hour interval.

**Table 4.** Postprandial patient-level RMSE^a^ and MAE^b^ obtained in predictions initiated at the time of meal intake or up to 15 minutes afterward, grouping the horizons into the 0-2 and 2- to 4-hour intervals.

Models	PH^c^=0-2 hours		PH=2-4 hours	
	RMSE (mg/dL)	MAE (mg/dL)	RMSE (mg/dL)	MAE (mg/dL)
Carbohydrates, mean (SD)	37.28 (10.1)	27.11 (7.7)	49.22 (15.4)	38.55 (12.0)
Carbohydrates+fats, mean (SD)	36.44 (9.8)	26.63 (7.8)	47.59 (14.2)	37.01 (11.3)
Carbohydrates+proteins, mean (SD)	36.83 (9.9)	26.97 (7.7)	48.09 (14.2)	37.50 (11.3)
Carbohydrates+sugars, mean (SD)	36.10 (9.7)	26.35 (7.5)	47.50 (13.8)	37.11 (10.8)
Carbohydrates+fats+proteins, mean (SD)	35.88 (9.4)	26.04 (7.0)	47.59 (13.5)	37.09 (10.4)
Carbohydrates+fats+proteins+sugars, mean (SD)	36.85 (10.5)	26.86 (8.0)	47.45 (15.8)	36.80 (12.6)
CC^d^+fats+proteins+sugars, mean (SD)	36.60 (10.2)	26.68 (7.7)	46.80(15.1)	36.29(12.1)

a RMSE: root mean squared error.

b MAE: mean absolute error.

c PH: prediction horizon.

d CC: complex carbohydrates.

Across all analyses, models incorporating additional nutritional information generally reduced both RMSE and MAE compared with the baseline, with greater improvement observed in the 2‐4 hours interval.

Table 5 presents the subject-level paired comparisons of RMSE between the carbohydrates model and the best-performing nutritional configuration at each prediction interval. The carbohydrates+fats+proteins model was compared with carbohydrates from 30 to 120 minutes, whereas the CC+fats+proteins+sugars model was compared with carbohydrates from 150 to 240 minutes. Holm correction was applied separately within the global and postprandial evaluations across the eight horizon-specific RMSE comparisons. In the global evaluation, the extended nutritional configurations yielded significantly lower RMSE values than the carbohydrates model at all evaluated horizons after Holm correction. All nominal 95% CI for the paired mean differences were also below zero. In the postprandial evaluation, all comparisons were nominally significant, and their nominal 95% CIs excluded zero. However, after Holm correction, statistical significance was retained only from 150 through 240 minutes.

**Table 5.** Subject-level paired comparisons of root mean squared error between the baseline carbohydrates model and the carbohydrates+fats+proteins model from 30 to 120 minutes or the complex carbohydrates+fats+proteins+sugars model from 150 to 240 minutes in the global and postprandial evaluations^a^.

PH^b^ (minutes)	Global			Postprandial		
	*P* value	95% CI	*P* Holm	*P* value	95% CI	*P* Holm
30	<.001	−0.70 to −0.21	.003	.02	−1.32 to −0.14	.12
60	<.001	−1.68 to −0.68	<.001	.01	−2.52 to −0.36	.08
90	<.001	−2.38 to −0.85	<.001	.03	[−3.20 to −0.22	.16
120	<.001	−2.78 to −1.01	<.001	.03	−3.69 to −0.18	.16
150	<.001	−2.98 to −1.36	<.001	.001	−4.49 to −1.23	.009
180	<.001	−3.06 to −1.32	<.001	.01	−3.64 to −0.51	.04
210	<.001	−3.07 to −1.09	<.001	.006	−3.32 to −0.61	.03
240	<.001	−3.09 to −0.93	.002	.004	−4.80 to −1.00	.03

a Nominal 95% CIs and unadjusted *P* values were obtained using paired Student *t*-tests. *P* values were adjusted for multiple comparisons using the Holm method.

b PH: prediction horizon.

The sensitivity analysis focused on exercise-free test windows included approximately 47% of the test set windows, and RMSE values were lower than global and postprandial evaluations. Nevertheless, the relative performance pattern was broadly preserved (Table 6). Carbohydrates+fats+proteins model achieved the lowest RMSE at 30, 60 and 90 minutes PH, whereas the CC+fats+proteins+sugars model obtained the lowest RMSE from 120 to 240 minutes. At 120 minutes, the CC+fats+proteins+sugars and carbohydrates+fats+proteins models produced similar values.

**Table 6.** Sensitivity analysis of model performance across prediction horizons using exercise-free test windows.^a^

Nutrients	PH^b^ (minutes)							
	30	60	90	120	150	180	210	240
Carbohydrates (mg/dL), mean (SD)	16.80 (5.1)	27.11 (9.0)	34.07 (12.3)	38.36 (14.7)	41.41 (16.0)	43.46 (17.3)	43.92 (17.1)	43.82 (16.7)
Carbohydrates+fats+proteins (mg/dL), mean (SD)	16.47 (4.5)	26.42 (8.5)	33.12 (12.4)	37.41 (14.5)	40.31 (15.5)	42.52 (16.6)	43.33 (16.4)	43.50 (16.2)
CC^c^+fats+proteins+sugars (mg/dL), mean (SD)	17.04 (5.6)	27.20 (10.1)	33.70 (13.8)	37.38 (15.5)	39.87 (15.8)	41.80 (16.7)	42.39 (16.3)	42.50 (16.6)

a Values are participant-level root mean squared errors, reported as mean (SD), in mg/dL.

b PH: prediction horizon.

c CC: complex carbohydrates.

EGA results are presented in Table 7. Across all PHs, more than 90% of the predictions generated by both models fell within the clinically acceptable zones A+B, ranging from 97.35% at 30 minutes to 92.82% at 240 minutes for the carbohydrates model and from 97.32% to 93.12% for the corresponding complete nutritional models. The complete nutritional models produced a higher percentage of predictions in Zone A at every PH. As the PH increased, the percentage of predictions in Zone A decreased, while the percentages in zones B and D increased. Predictions in zones C and E remained below 0.5% for all models and horizons.

**Table 7.** Distribution (%) of glucose predictions across the Clarke error grid analysis zones for the baseline model (carbohydrates) and the nutritional model achieving the lowest root mean squared error at each PH^a^: carbohydrates+fats+proteins for PHs of 30-120 minutes and CC^b^+fats+proteins+sugars for PHs of 150-240 minutes.

PH (minutes) and model	Zone A (%)	Zone B (%)	Zone C (%)	Zone D (%)	Zone E (%)
30					
Carbohydrates	86.76	10.59	0.02	2.62	0.00
Carbohydrates+fats+proteins	87.17	10.15	0.01	2.66	0.00
60					
Carbohydrates	71.58	24.45	0.11	3.79	0.06
Carbohydrates+fats+proteins	72.80	23.32	0.08	3.77	0.04
90					
Carbohydrates	61.53	33.32	0.25	4.76	0.14
Carbohydrates+fats+proteins	63.57	31.56	0.12	4.68	0.07
120					
Carbohydrates	55.73	38.19	0.38	5.56	0.14
Carbohydrates+fats+proteins	58.00	36.19	0.14	5.60	0.08
150					
Carbohydrates	52.69	40.56	0.43	6.20	0.12
CC+fats+proteins+sugars	55.47	38.13	0.22	6.08	0.10
180					
Carbohydrates	50.93	42.05	0.44	6.45	0.13
CC+fats+proteins+sugars	53.79	39.58	0.28	6.26	0.09
210					
Carbohydrates	49.84	43.08	0.34	6.61	0.13
CC+fats+proteins+sugar	53.14	40.08	0.31	6.38	0.09
240					
Carbohydrates	49.23	43.59	0.33	6.75	0.10
CC+fats+proteins+sugars	52.56	40.56	0.31	6.43	0.13

a PH: prediction horizon.

b CC: complex carbohydrates.

**Figure 4.**
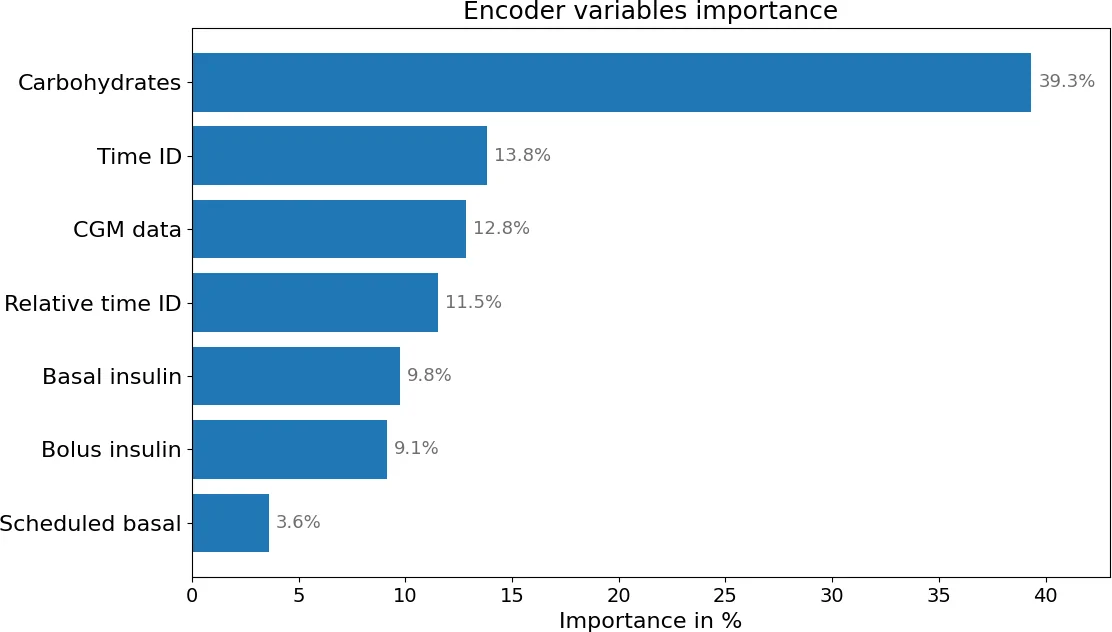
Encoder variable importance estimates from temporal fusion transformer models trained on CGM, insulin, and meal composition data (carbohydrates model) in adults from the type 1 diabetes and exercise initiative. CGM: continuous glucose monitoring.

**Figure 5.**
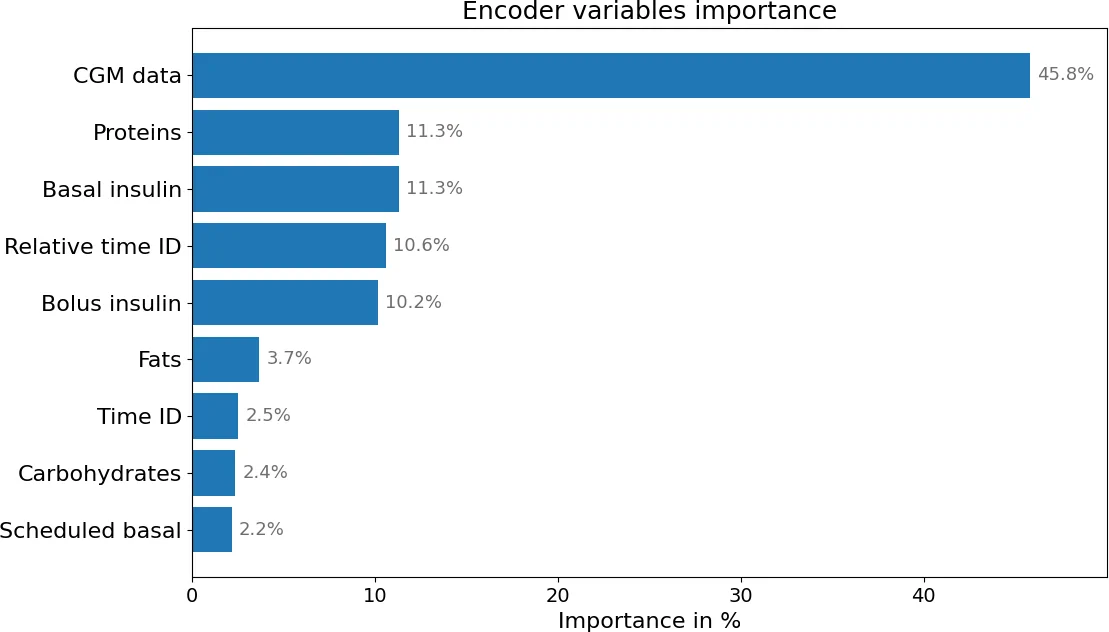
Encoder variable importance estimates from temporal fusion transformer models trained on CGM, insulin, and meal composition data (carbohydrates+fats+proteins model) in adults from the type 1 diabetes and exercise initiative study. The bar plot shows the relative contribution of nutritional and nonnutritional variables for a configuration including carbohydrates, fats, and proteins. CGM: continuous glucose monitoring.

**Figure 6.**
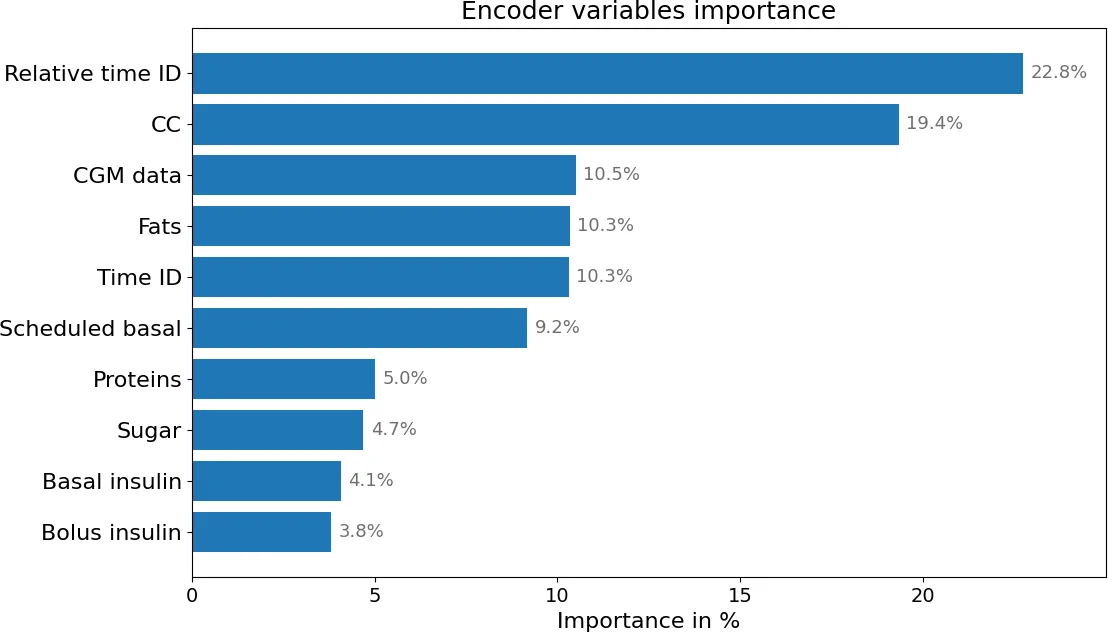
Encoder variable importance estimates from temporal fusion transformer models trained on CGM, insulin, and meal composition data (CC+fats+proteins+sugars model) in adults from the type 1 diabetes and exercise initiative study. The bar plot shows the relative contribution of nutritional and nonnutritional variables for a configuration distinguishing complex carbohydrates and sugars together with fats and proteins. CGM: continuous glucose monitoring

The following figures illustrate the importance of the TFT encoder variables for the selected configurations. In the baseline model (Figure 4), carbohydrates were the most influential variable, as expected. In the carbohydrates+fats+proteins model (Figure 5), which performed best at the shortest PH, CGM data was the most important feature overall, followed by proteins and basal insulin. Among nutritional inputs, proteins showed the highest relative importance, whereas carbohydrates and fats showed a lower contribution. In contrast, in the CC+fats+proteins+sugars model (Figure 6), which achieved the best performance at longer PHs, CC emerged as the most relevant nutritional variable, with fats contributing substantially, while proteins and sugars showed lower importance.

**Figure 7.**
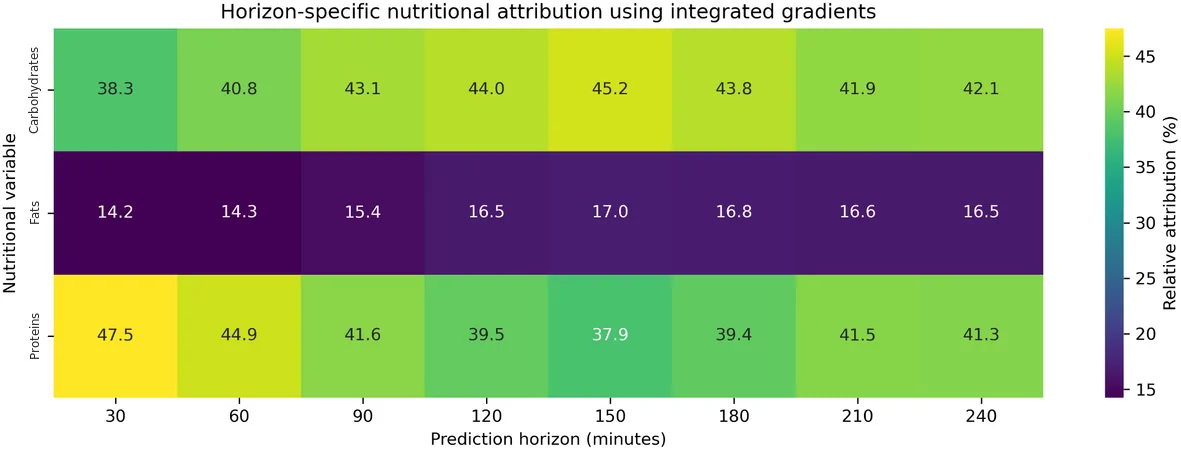
Horizon-specific relative absolute integrated gradients attributions of nutritional inputs in the carbohydrates+fats+proteins temporal fusion transformer model. The analysis was conducted on a participant-balanced sample of postprandial test windows from adults in the type 1 diabetes and exercise initiative cohort, with up to 20 windows selected per participant. Integrated gradients were calculated using a 0-g nutritional baseline while retaining the remaining inputs unchanged. Absolute attributions were aggregated across encoder time steps and normalized within each prediction horizon to sum to 100%. Values represent the relative magnitude of model dependence and not the direction or causal effect of each nutrient.

The horizon-specific integrated gradients analysis showed partial agreement with the aggregate VSN weights while providing additional horizon-dependent information. In the carbohydrates+fats+proteins model, the VSN identified proteins as the nutritional variable with the highest aggregate selection weight. Consistently, proteins had the highest integrated gradients attribution (Figure 7) at the 30- and 60-minute PHs. However, carbohydrates exceeded proteins from 90 minutes onward, despite their relatively low aggregate VSN weight. Fats showed the lowest integrated gradients attribution at all horizons, whereas their aggregate VSN weight was higher than that of carbohydrates.

**Figure 8.**
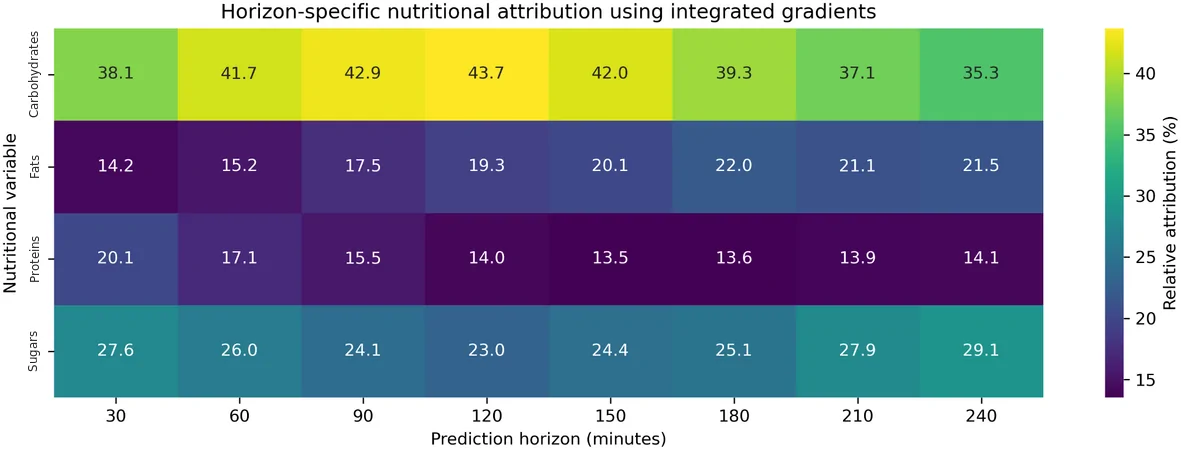
Horizon-specific relative absolute integrated gradients attributions of nutritional inputs in the CC+fats+proteins+sugars temporal fusion transformer model. The analysis was conducted on a participant-balanced sample of postprandial test windows from adults in the type 1 diabetes and exercise initiative cohort, with up to 20 windows selected per participant. Integrated gradients were calculated using a 0-g nutritional baseline while retaining the remaining inputs unchanged. Absolute attributions were aggregated across encoder time steps and normalized within each prediction horizon to sum to 100%. Values represent the relative magnitude of model dependence and not the direction or causal effect of each nutrient. CC: complex carbohydrates.

In the CC+fats+proteins+sugars model (Figure 8), both methods identified CC as the dominant nutritional variable. Nevertheless, the ranking of the remaining nutritional variables differed. Fats had the second-highest nutritional VSN weight, whereas sugars consistently showed the second-highest integrated gradients attribution. Fat attribution increased with the PH and exceeded protein attribution from 90 minutes onward, while protein attribution progressively decreased.

## Discussion

### Principal Results

The inclusion of additional nutritional variables improved predictive capacity compared with the carbohydrate-only baseline, suggesting that relying exclusively on carbohydrates may not fully capture the dietary information relevant to glucose forecasting.

At shorter PHs, the combination of carbohydrates, fats, and proteins yielded the lowest prediction errors. Although explicit information on sugars could be expected to improve short-term predictions due to their rapid absorption, their inclusion did not lead to consistent improvements. The carbohydrates+sugars model produced similar performance to other configurations, whereas adding sugars alongside all nutritional variables occasionally increased prediction error. This pattern suggests that sugar content may provide limited independent information when total carbohydrate content is already included.

The subject-level statistical analysis confirmed that the reductions in RMSE observed in the global evaluation were consistent across participants, as all comparisons remained statistically significant after Holm correction. In the postprandial evaluation, lower RMSE values were also observed at all PHs; however, the differences from 30 to 120 minutes did not remain significant after correction for multiple comparisons. Therefore, the apparent advantage of the carbohydrates+fats+proteins model during the early postprandial period should be interpreted cautiously. In contrast, the differences obtained by the CC+fats+proteins+sugars model remained significant from 150 to 240 minutes, providing stronger evidence for the value of a more detailed nutritional representation during the later postprandial phase. The absence of adjusted significance at earlier horizons should not be interpreted as evidence of equivalence between models. The smaller number of eligible postprandial windows and greater interindividual variability may have contributed to the wider confidence intervals and reduced statistical power in this analysis.

The horizon-dependent pattern is consistent with known physiological mechanisms of the postprandial response. Fats can delay gastric emptying, while fats and proteins can modulate glucose absorption. Similarly, CC contribute to sustained glycemic responses over time. The results indicate that the contribution of nutritional variables is not uniform across the postprandial period and becomes increasingly informative at longer PHs.

In the evaluation of postprandial windows, similar trends were observed. In particular, the incorporation of sugars did not improve short-term performance and, in some cases, slightly increased prediction error, especially at the 30-minute PH. This finding further supports the idea that sugars may not provide additional independent information beyond total carbohydrate content when both variables are included simultaneously. In contrast, separating sugars from CC may enable a more precise representation of meal composition, which likely explains the improvements observed at longer horizons.

Overall, these findings suggest that a more comprehensive nutritional representation may contribute to postprandial glucose forecasting. The carbohydrates+fats+proteins configuration showed the lowest errors in early PH, whereas the most detailed CC+fats+proteins+sugars configuration showed the lowest error at longer PHs. These results indicate that different representations of meal composition may provide useful predictive information at different forecast horizons, although they should not be interpreted as evidence of independent or causal physiological effects. Lower RMSE values in exercise-free test windows suggest less challenging forecasting conditions. However, half of the original test windows were excluded, producing a smaller subset with a potentially different distribution of glucose trajectories and clinical events, suggested by larger standard deviations. More importantly, the relative performance pattern was broadly maintained: carbohydrates+fats+proteins achieved the lowest errors at early horizons, whereas CC+fats+proteins+sugars performed better at later horizons.

Although the reductions in RMSE were statistically significant in several comparisons, statistical significance does not necessarily imply clinical relevance. EGA complemented the conventional regression metrics by providing information about the potential clinical consequences of prediction errors. More than 90% of predictions remained within the clinically acceptable zones A and B, even at the longest PH. The inclusion of complete nutritional information resulted in a modest increase in the proportion of Zone A predictions and generally reduced the proportion of predictions outside zones A and B. Nevertheless, the reduction in Zone A and the progressive increase in Zone D as the PH increased indicate lower clinical reliability at longer horizons. This finding should be considered when predictions are used to support therapeutic decisions. Moreover, although error grid analysis provides a clinically oriented assessment of pointwise prediction errors, it does not account for temporal glucose dynamics or constitute prospective evidence of clinical safety.

The interpretability analyses of the TFT provided additional information to contextualize the performance results. In the carbohydrates model, carbohydrates dominated the contribution of nutritional variables, likely because they were the only available dietary variable and therefore represented most of the meal-related information. When additional nutritional variables were incorporated, the aggregate VSN weights were redistributed across nutrients and CGM became dominant. This shift may reflect the role of CGM data as a direct representation of the current glycemic state, while meal-related information previously concentrated in carbohydrates became distributed among several nutrients. Integrated gradients patterns indicate that the model’s dependence on individual nutritional input varies across the forecast horizon. The attribution assigned to complete nutritional information at longer horizons provides context for the performance improvements observed during the 2‐4-hour postprandial interval, but it does not establish that any individual nutrient caused these improvements.

The VSN and integrated gradients analyses showed only partial agreement. Both approaches highlighted proteins at short horizons in the carbohydrates+fats+proteins model and identified CC as the dominant nutritional input in the CC+fats+proteins+sugars model. However, they differed in the ranking of other variables. In particular, fats had the second-highest nutritional VSN weight in the CC+fats+proteins+sugars model, whereas sugars had the second-highest integrated gradients attribution. These differences are expected because VSN weights represent the model’s internal variable-selection mechanism aggregated across samples and time steps, whereas integrated gradients quantify output-specific sensitivity relative to a predefined baseline in selected postprandial windows. Correlations among nutritional variables may also cause attribution to be distributed differently across inputs.

Overall, the performance results support the additional predictive value of detailed nutritional information in this dataset, whereas the interpretability analyses indicate how the trained models internally used these variables. Neither VSN weights nor integrated gradients establish causal, independent, or physiological effects of individual nutrients. Furthermore, because absolute integrated gradients were used, the reported percentages represent attribution magnitude and not the direction of the nutrient’s effect on predicted glucose.

### Comparison With Prior Work

The comparison with previous studies should be interpreted with caution, due to differences in the PHs evaluated, the amount and distribution of the data used, the evaluation protocol, and the specific objective of the model, which may vary between continuous glucose prediction and the classification of postprandial events. Calzavara et al [33] reported recent results for glucose prediction models, with typical RMSE values for a 30-minute horizon between 17 and 21 mg/dL. In the present study, the best global model achieved RMSE values of 18.53 mg/dL at the 30-minute horizon, which falls within this previously reported range. Similarly, a systematic review conducted by Zhu et al [8] also reported results for the 60-minute horizon with typical RMSE values between 19.90 and 38.00 mg/dL. In the present study, the RMSE at 60 minutes (29.22 mg/dL) also lies within the range reported in previous studies.

The most thematically relevant comparison can be established with the works by Annuzzi et al [5,21], as these studies focused on postprandial glucose prediction incorporating nutritional information beyond carbohydrates. In the study published in 2023, Annuzzi et al [5] reported RMSE values of 8.68 and 24.15 mg/dL at PHs of 30 and 60 minutes, respectively. Subsequently, in their 2024 study [21], they reported RMSE values of 24.74 and 50.15 mg/dL for PHs of 60 and 120 minutes, respectively. Although these values provide a useful reference, direct comparison is limited by differences between datasets, cohorts, and evaluation protocols. Consequently, the numerical differences between the results in this study and their results cannot be attributed specifically to the forecasting architecture or the nutritional representation.

However, a qualitative comparison regarding explainability can be established. Annuzzi et al [21] analyzed feature relevance across different PHs and reported horizon-dependent differences in the contribution of nutritional variables. In their study, fats showed increased relevance at the 15-minute PH compared with other nutritional variables, whereas carbohydrates became more relevant at the 60- and 120-minute PHs. The horizon-specific integrated gradients analysis conducted in the present study similarly showed that the model’s dependence on individual nutrients varies across the PH. However, the ranking differed between nutritional configurations and between integrated gradients and VSN weights. These differences may reflect the nutritional variables provided to each model, the correlations among nutrients, the analyzed samples, and the distinct meanings of the attribution methods. Therefore, similarities or differences in nutrient relevance across studies should be interpreted as model-specific patterns rather than evidence of general physiological effects.

Therefore, findings in this study suggest that the added nutritional variables influence model predictions by providing complementary information across different postprandial horizons. From a clinical perspective, this supports the idea that postprandial glucose forecasting models may benefit from representing meals beyond total carbohydrate intake. Although the present study does not provide therapeutic recommendations, the horizon-dependent contribution of nutritional variables could inform future clinical decision-support tools for meal planning, postprandial monitoring, or retrospective review of glycemic excursions. In particular, interpretable models may help identify whether early or delayed postprandial predictions are more influenced by macronutrient composition, supporting a more individualized analysis of meal-related glucose dynamics.

Recent forecasting architectures, including N-HiTS, PatchTST, TimeMixer, TimesNet, Informer, and Autoformer, constitute relevant alternatives to the TFT for long-horizon time-series prediction. However, the present study was designed to evaluate the incremental contribution of nutritional composition within a fixed forecasting architecture rather than to provide a comprehensive architectural benchmark.

### Limitations

This study has some limitations. First, meals were documented with a study-specific mobile application and reviewed by a trained human rater to identify the foods depicted and map them to the appropriate nutrient entries and estimate meal composition. Although this procedure yielded more structured and standardized nutritional information than traditional self-report methods, it should be noted that nutritional estimates derived from photographs, participant reports, and post hoc dietary analysis are still subject to measurement error. Such errors may attenuate the apparent contribution of specific nutrients, introduce noise into model training, and affect the stability of interpretability analyses.

Each model configuration was trained once using a fixed random seed. Although this ensured a consistent within-study comparison across configurations, variability attributable to model initialization and stochastic optimization was not quantified. The reported differences should therefore be interpreted as results from a controlled single-run comparison, and their stability across independent training runs remains to be assessed. In addition, the analysis was conducted using a single patient-wise split of the T1-DEXI cohort into training, validation, and test sets. This design minimizes the risk of data leakage, as model performance is evaluated on previously unseen participants. However, validation on an independent dataset is still required to assess generalizability and capture the full range of variability that may be encountered across different clinical settings. Such validation was not feasible because we did not have access to a public dataset that currently provides continuous glucose monitoring, insulin records, and detailed meal composition under real-world conditions.

Additionally, the included cohort had a more favorable HbA1c distribution than the excluded cohort (*P*=.004), indicating that participants with better glycemic control were overrepresented. This difference may limit the generalizability of the findings to individuals with poorer glycemic control.

Another limitation concerns missing CGM data in the validation and test sets. Input gaps shorter than 30 minutes were imputed using LOCF to avoid future information leakage. Although imputed observations were infrequent, LOCF may attenuate rapid glycemic changes, particularly postprandially, potentially increasing errors in affected windows. Therefore, a modest impact on performance cannot be excluded.

Although fiber information was available, it was not evaluated as an independent input, and the derived CC variable may include fiber and other nonsugar carbohydrate fractions. Consequently, the predictive contribution associated with CC cannot be interpreted independently of these components.

Although an oversampling strategy was used to increase the representation of postprandial periods during training, the number of samples with detailed nutritional information remained limited compared with the overall amount of glucose data, accounting for approximately 26,000 out of 254,000 windows. This imbalance may have affected the model’s ability to fully capture the contribution of nutritional variables.

Finally, VSN and integrated gradients provide complementary but noncausal insights into model behavior. VSN weights are normalized within each model and aggregated across samples and time, precluding direct comparisons between configurations or interpretation as effect sizes. Integrated gradients depend on the selected baseline and analyzed windows, while absolute attributions indicate magnitude but not direction. Moreover, correlations may redistribute importance across inputs. Therefore, neither method isolates causal, independent, or physiological effects of individual nutrients.

### Future Work

Future work should compare the TFT with more recent forecasting architectures, including N-HiTS, PatchTST, TimeMixer, TimesNet, Informer, and Autoformer, using identical participant-level partitions, input variables, prediction horizons, and hyperparameter-optimization procedures. Such comparisons would enable a more rigorous assessment of the relative performance of these architectures.

External validation should also be performed when datasets combining CGM data with sufficiently detailed nutritional records become available. This would help determine the generalizability of the findings across different populations and data-collection settings.

Finally, future studies could investigate whether additional dietary components provide incremental predictive information. Potential candidates include dietary fiber and distinctions among different types of fat. Other nutrients with a less direct relationship with acute glycemic responses could also be explored when supported by an appropriate physiological rationale and sufficient data availability.

### Conclusions

This study evaluated whether incorporating detailed nutritional information beyond carbohydrates provides additional predictive value for glucose prediction models. The models trained with additional variables related to meal composition generally achieved modest, although sometimes statistically significant, reductions in prediction errors, with the clearest differences at longer horizons. The carbohydrates, fats, and proteins model achieved lower errors at shorter horizons, while the model containing CC, fats, proteins, and sugars performed better at longer horizons.

The interpretability mechanism provided by VSN relative weights and integrated gradients analysis suggested that contributions of nutritional variables were horizon-dependent, although these attributions should not be interpreted as causal or physiological effects. Overall, these findings suggest the importance of considering a more comprehensive nutritional characterization in the development of postprandial glucose prediction models. These results also may inform future research on nutrition-aware glucose forecasting and support the development of more interpretable prediction models. However, additional external validation and prospective evaluations are required before assessing potential clinical utility.

## Supplementary material

10.2196/105049 Multimedia Appendix 1 Demographic and treatment characteristics of adults with type 1 diabetes from the T1-DEXI study cohort across the training, validation, test, and excluded subsets.

10.2196/105049 Multimedia Appendix 2 Hyperparameter search space explored using grid search during model development.

## Abbreviations

CC: complex carbohydrates
CGM: continuous glucose monitoring
EGA: error grid analysis
GPFormer: Glucose Prediction Transormer
IRB: institutional review board
LOCF: last-observation-carried-forward
LSTM: long short-term memory
MAE: mean absolute error
N-BEATS: Neural-Basis Expansion Analysis Time Series
N-HiTS: Neural Hierarchical Interpolation for Time Series
PatchTST: Patch Time Series Transformer
PH: prediction horizon
RMSE: root mean squared error
SHAP: Shapley additive explanations
T1-DEXI: type 1 diabetes and exercise initiative
T1D: type 1 diabetes
TFT: temporal fusion transformer
VSN: variable selection network
