# Supplementary material for: Interpretable Multihorizon Glucose Forecasting for Assessing Nutritional Information Impact in Type 1 Diabetes Management: Model Development and Validation Study
Source: JMIR AI. 2026 Sep 21;5:e105049. doi: 10.2196/105049 (PMC13592460; doi:10.2196/105049)
Supplement: Supplementary file 1 [file ai-v5-e105049-s001.docx]

Table S1. Demographic and treatment characteristics of adults with type 1 diabetes from the T1-DEXI study cohort across the included subsets (training, validation, test), and the excluded subset. Values are reported as the number of participants (n) and percentage within each subset. Included and excluded participants were compared using Fisher exact tests for sex and ethnicity and Fisher–Freeman–Halton exact tests for treatment modality, age group, HbA1c category, and type 1 diabetes duration.

|  | **Training**  **(N=281)** | **Validation**  **(N=35)** | **Test**  **(N=35)** | **Included**  **(N=351)** | **Excluded**  **(N=48)** | ***P* value** |
| --- | --- | --- | --- | --- | --- | --- |
|  | **n (%)** | **n (%)** | **n (%)** | **n (%)** | **n (%)** |  |
| **Female** | 205 (73.0) | 23 (65.7) | 24 (68.6) | 252 (71.8) | 39 (81.3) | .22 |
| **Male** | 76 (27.0) | 12 (34.3) | 11 (31.4) | 99 (28.2) | 9 (18.7) |  |
| **Closed loop** | 143 (50.9) | 19 (54.3) | 20 (57.1) | 182 (51.9) | 21 (43.7) | .16 |
| **Insulin Pump** | 126 (44.8) | 13 (37.1) | 11 (31.5) | 150 (42.7) | 21 (43.7) |  |
| **Multiple Daily injections** | 12 (4.3) | 3 (8.6) | 4 (11.4) | 19 (5.4) | 6 (12.5) |  |
| **Age 18-29 years** | 106 (37.7) | 19 (54.3) | 15 (42.9) | 140 (39.9) | 28 (58.3) | .10 |
| **Age 30-39 years** | 73 (26.0) | 4 (11.4) | 11 (31.4) | 88 (25.1) | 6 (12.5) |  |
| **Age 40-49 years** | 46 (16.4) | 5 (14.3) | 3 (8.6) | 54 (15.4) | 8 (16.7) |  |
| **Age 50-59 years** | 28 (10.0) | 4 (11.4) | 2 (5.7) | 34 (9.7) | 4 (8.3) |  |
| **Age >59 years** | 28 (10.0) | 3 (8.6) | 4 (11.4) | 35 (10.0) | 2 (4.2) |  |
| **White** | 244 (86.8) | 33 (94.3) | 30 (85.7) | 307 (87.5) | 40 (83.3) | .49 |
| **Other ethnic groups** | 37 (13.2) | 2 (5.7) | 5 (14.3) | 44 (12.5) | 8 (16.7) |  |
| **HbA1c <7%** | 199 (70.8) | 24 (68.6) | 25 (71.4) | 248 (70.6) | 25 (52.1) | .004 |
| **HbA1c 7% TO <8%** | 70 (24.9) | 11 (31.4) | 8 (22.9) | 89 (25.4) | 16 (33.3) |  |
| **HbA1c ≥8%** | 12 (4.3) | 0 (0) | 2 (5.7) | 14 (4.0) | 7 (14.6) |  |
| **T1D Duration <5 years** | 27 (9.6) | 2 (5.7) | 4 (11.4) | 33 (9.4) | 7 (14.6) | .54 |
| **T1D Duration 5 to ≤10 years** | 48 (17.1) | 5 (14.3) | 7 (20.0) | 60 (17.1) | 7 (14.6) |  |
| **T1D Duration >10 years** | 206 (73.3) | 28 (80.0) | 24 (68.6) | 258 (73.5) | 34 (70.8) |  |

***HbA1c:*** *glycated hemoglobin*
