# Supplementary material for: Interpretable Multihorizon Glucose Forecasting for Assessing Nutritional Information Impact in Type 1 Diabetes Management: Model Development and Validation Study
Source: JMIR AI. 2026 Sep 21;5:e105049. doi: 10.2196/105049 (PMC13592460; doi:10.2196/105049)
Supplement: Supplementary file 2 [file ai-v5-e105049-s002.docx]

Table S1. Hyperparameter search space explored using grid search, fixed hyperparameters, and selected values for the Temporal Fusion Transformer model.

| Hyperparameters | Search space | Selected value |
| --- | --- | --- |
| Attention heads | {4, 6} | 4 |
| Hidden units | {64, 128} | 64 |
| Learning rate | {$1\times{10}^{-3}$, 5 $\times{10}^{-4}$, $1\times{10}^{-4}$} | $1\times{10}^{-4}$ |
| Dropout | Prespecified and fixed | 0.2 |
| Recurrent layers | Prespecified and fixed | 1 |
| Quantiles | Prespecified and fixed | [0.25, 0.5, 0.75] |
| Optimizer | Prespecified and fixed | Adam |
| Batch size | Prespecified and fixed | 64 |
| Max epochs | Prespecified and fixed | 100 |
| Patience | Prespecified and fixed | 10 |
